# Supplementary material for: CREPT is required for murine stem cell maintenance during intestinal regeneration
Source: Nat Commun. 2021 Jan 11;12:270. doi: 10.1038/s41467-020-20636-9 (PMC7801528; doi:10.1038/s41467-020-20636-9)
Supplement: Supplementary file 1 — Supplementary Information [file 41467_2020_20636_MOESM1_ESM.pptx]

## Slide 1
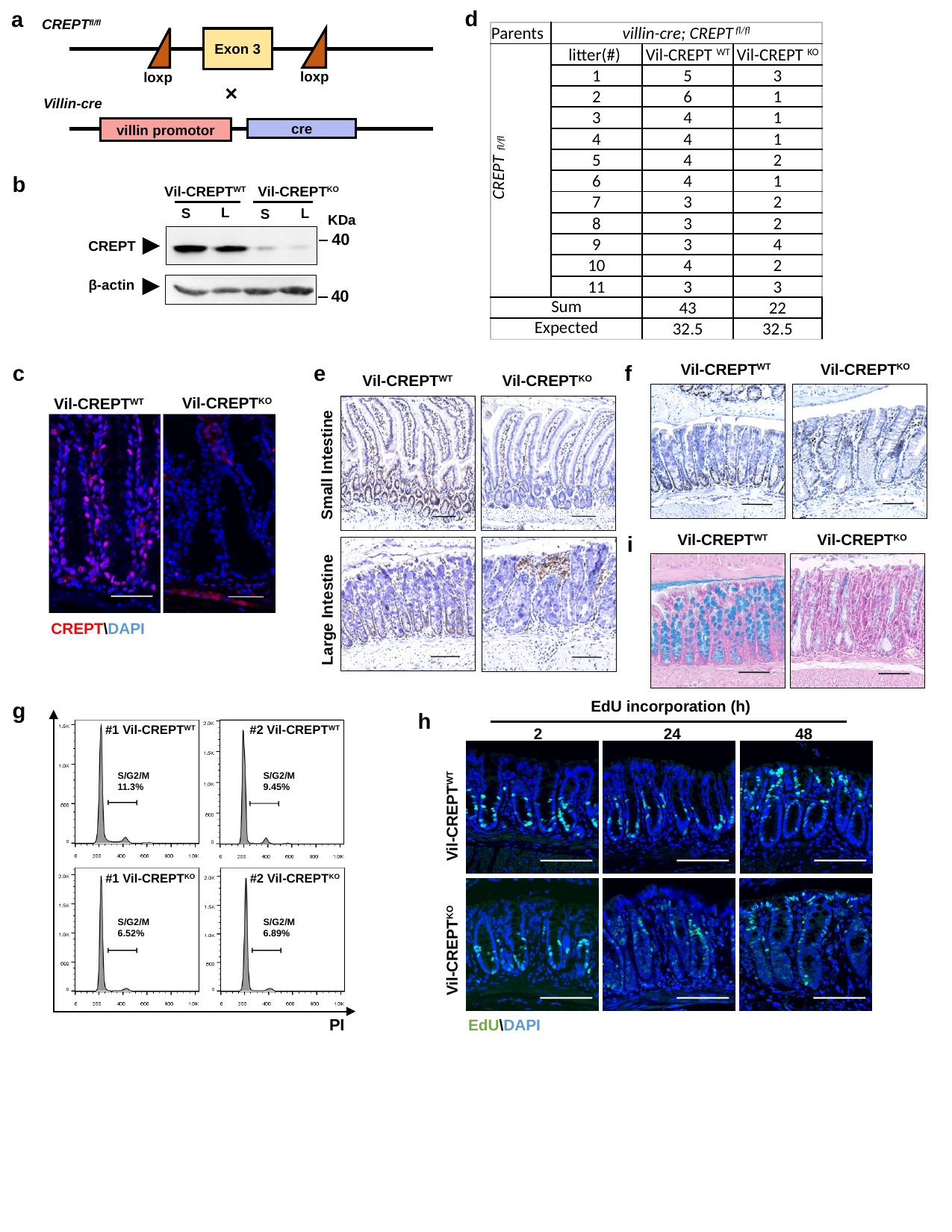

d
a
CREPTfl/fl
Exon 3
loxp
loxp
×
Villin-cre
villin promotor
cre
| Parents | villin-cre; CREPT fl/fl | | |
| --- | --- | --- | --- |
| CREPTfl/fl | litter(#) | Vil-CREPT WT | Vil-CREPT KO |
| | 1 | 5 | 3 |
| | 2 | 6 | 1 |
| | 3 | 4 | 1 |
| | 4 | 4 | 1 |
| | 5 | 4 | 2 |
| | 6 | 4 | 1 |
| | 7 | 3 | 2 |
| | 8 | 3 | 2 |
| | 9 | 3 | 4 |
| | 10 | 4 | 2 |
| | 11 | 3 | 3 |
| Sum | | 43 | 22 |
| Expected | | 32.5 | 32.5 |
b
Vil-CREPTWT
Vil-CREPTKO
L
L
S
S
CREPT
β-actin
KDa
40
40
c
e
Vil-CREPTKO
Vil-CREPTWT
f
Vil-CREPTKO
Vil-CREPTWT
Small Intestine
Large Intestine
Vil-CREPTKO
Vil-CREPTWT
CREPT\DAPI
Vil-CREPTKO
Vil-CREPTWT
i
EdU incorporation (h)
2
24
48
Vil-CREPTWT
Vil-CREPTKO
EdU\DAPI
g
#1 Vil-CREPTWT
#2 Vil-CREPTWT
S/G2/M
11.3%
S/G2/M
9.45%
#1 Vil-CREPTKO
#2 Vil-CREPTKO
S/G2/M
6.52%
S/G2/M
6.89%
PI
h

## Slide 2
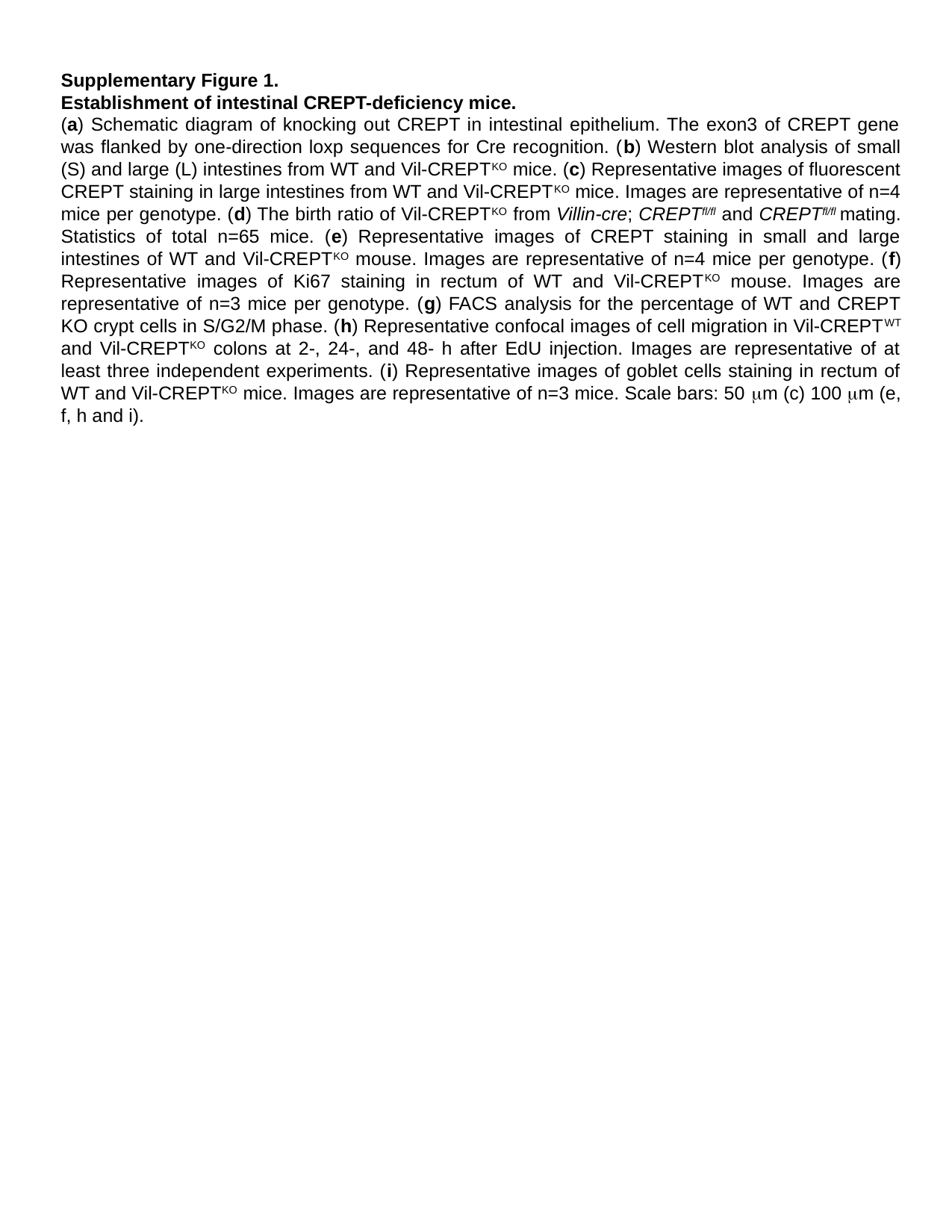

Supplementary Figure 1.
Establishment of intestinal CREPT-deficiency mice.
(a) Schematic diagram of knocking out CREPT in intestinal epithelium. The exon3 of CREPT gene was flanked by one-direction loxp sequences for Cre recognition. (b) Western blot analysis of small (S) and large (L) intestines from WT and Vil-CREPTKO mice. (c) Representative images of fluorescent CREPT staining in large intestines from WT and Vil-CREPTKO mice. Images are representative of n=4 mice per genotype. (d) The birth ratio of Vil-CREPTKO from Villin-cre; CREPTfl/fl and CREPTfl/fl mating. Statistics of total n=65 mice. (e) Representative images of CREPT staining in small and large intestines of WT and Vil-CREPTKO mouse. Images are representative of n=4 mice per genotype. (f) Representative images of Ki67 staining in rectum of WT and Vil-CREPTKO mouse. Images are representative of n=3 mice per genotype. (g) FACS analysis for the percentage of WT and CREPT KO crypt cells in S/G2/M phase. (h) Representative confocal images of cell migration in Vil-CREPTWT and Vil-CREPTKO colons at 2-, 24-, and 48- h after EdU injection. Images are representative of at least three independent experiments. (i) Representative images of goblet cells staining in rectum of WT and Vil-CREPTKO mice. Images are representative of n=3 mice. Scale bars: 50 mm (c) 100 mm (e, f, h and i).

## Slide 3
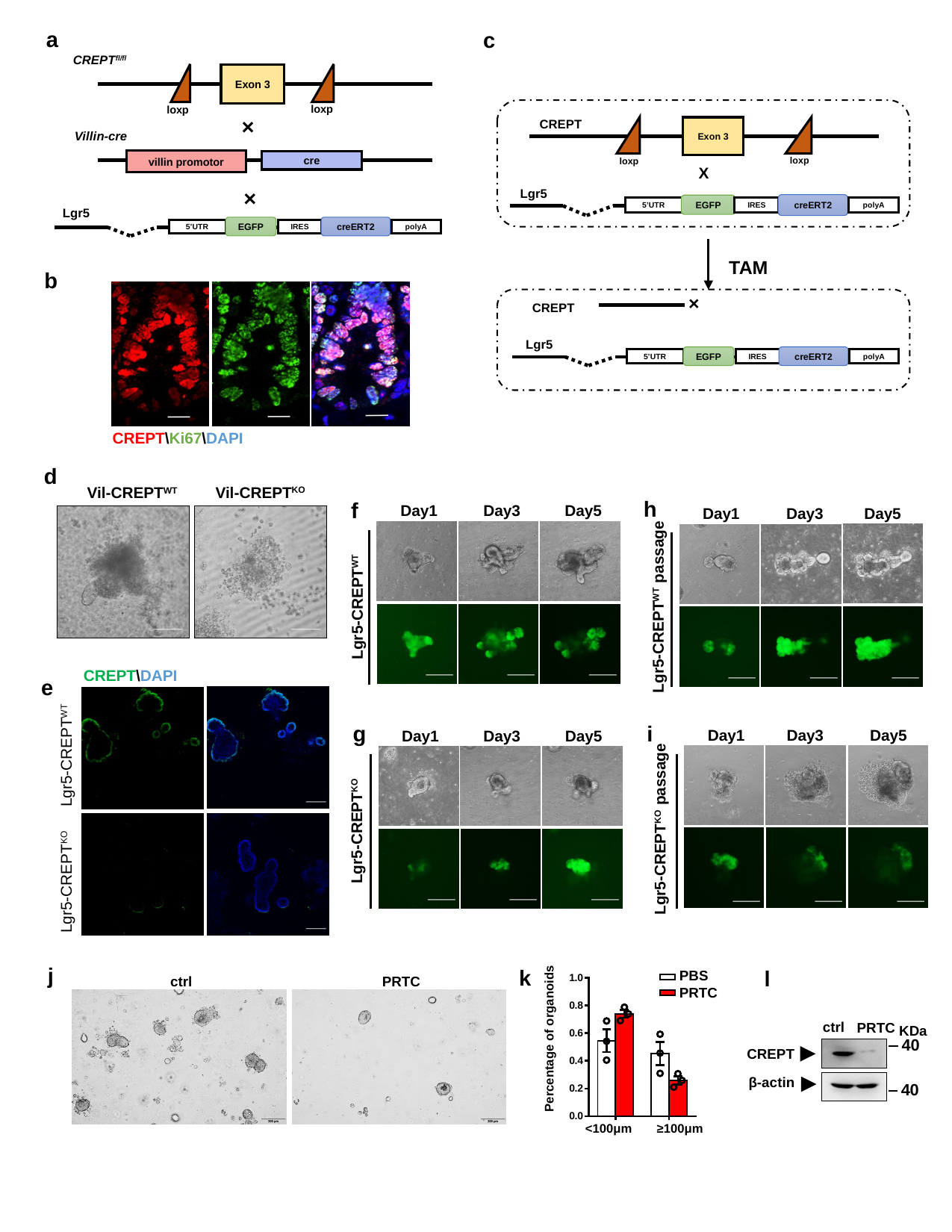

a
c
CREPTfl/fl
Exon 3
loxp
loxp
×
Villin-cre
villin promotor
cre
CREPT
Exon 3
loxp
loxp
X
Lgr5
creERT2
EGFP
polyA
5’UTR
IRES
TAM
×
CREPT
Lgr5
EGFP
creERT2
polyA
5’UTR
IRES
×
Lgr5
EGFP
creERT2
polyA
5’UTR
IRES
b
CREPT\Ki67\DAPI
d
Vil-CREPTKO
Vil-CREPTWT
h
f
Day1
Day3
Day5
Lgr5-CREPTWT
Day1
Day3
Day5
Lgr5-CREPTWT passage
CREPT\DAPI
Lgr5-CREPTWT
Lgr5-CREPTKO
e
g
i
Day1
Day3
Day5
Lgr5-CREPTKO passage
Day1
Day3
Day5
Lgr5-CREPTKO
j
k
PBS
Percentage of organoids
PRTC
≥100μm
<100μm
l
ctrl
PRTC
ctrl
PRTC
CREPT
β-actin
KDa
40
40

## Slide 4
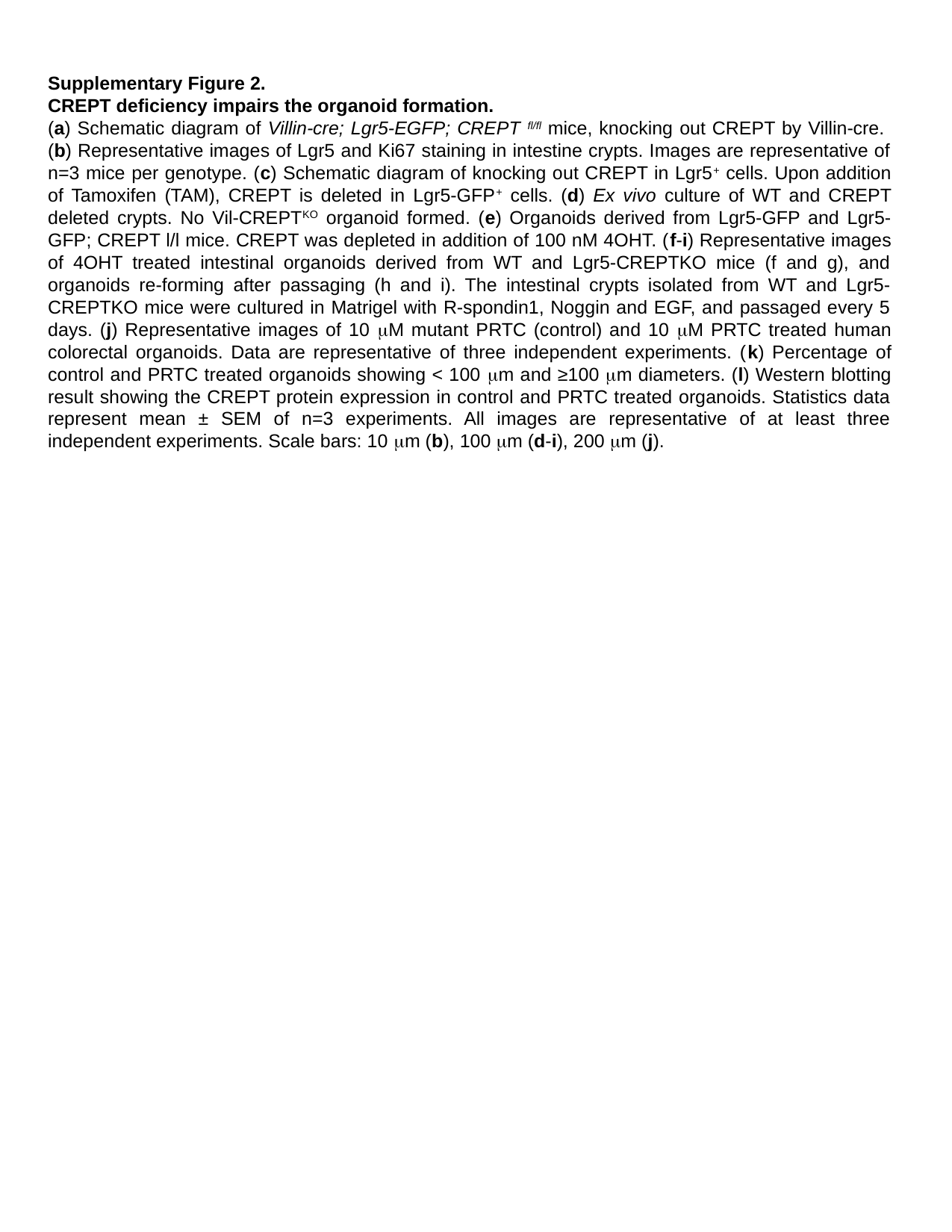

Supplementary Figure 2.
CREPT deficiency impairs the organoid formation.
(a) Schematic diagram of Villin-cre; Lgr5-EGFP; CREPT fl/fl mice, knocking out CREPT by Villin-cre. (b) Representative images of Lgr5 and Ki67 staining in intestine crypts. Images are representative of n=3 mice per genotype. (c) Schematic diagram of knocking out CREPT in Lgr5+ cells. Upon addition of Tamoxifen (TAM), CREPT is deleted in Lgr5-GFP+ cells. (d) Ex vivo culture of WT and CREPT deleted crypts. No Vil-CREPTKO organoid formed. (e) Organoids derived from Lgr5-GFP and Lgr5-GFP; CREPT l/l mice. CREPT was depleted in addition of 100 nM 4OHT. (f-i) Representative images of 4OHT treated intestinal organoids derived from WT and Lgr5-CREPTKO mice (f and g), and organoids re-forming after passaging (h and i). The intestinal crypts isolated from WT and Lgr5-CREPTKO mice were cultured in Matrigel with R-spondin1, Noggin and EGF, and passaged every 5 days. (j) Representative images of 10 mM mutant PRTC (control) and 10 mM PRTC treated human colorectal organoids. Data are representative of three independent experiments. (k) Percentage of control and PRTC treated organoids showing < 100 mm and ≥100 mm diameters. (l) Western blotting result showing the CREPT protein expression in control and PRTC treated organoids. Statistics data represent mean ± SEM of n=3 experiments. All images are representative of at least three independent experiments. Scale bars: 10 mm (b), 100 mm (d-i), 200 mm (j).

## Slide 5
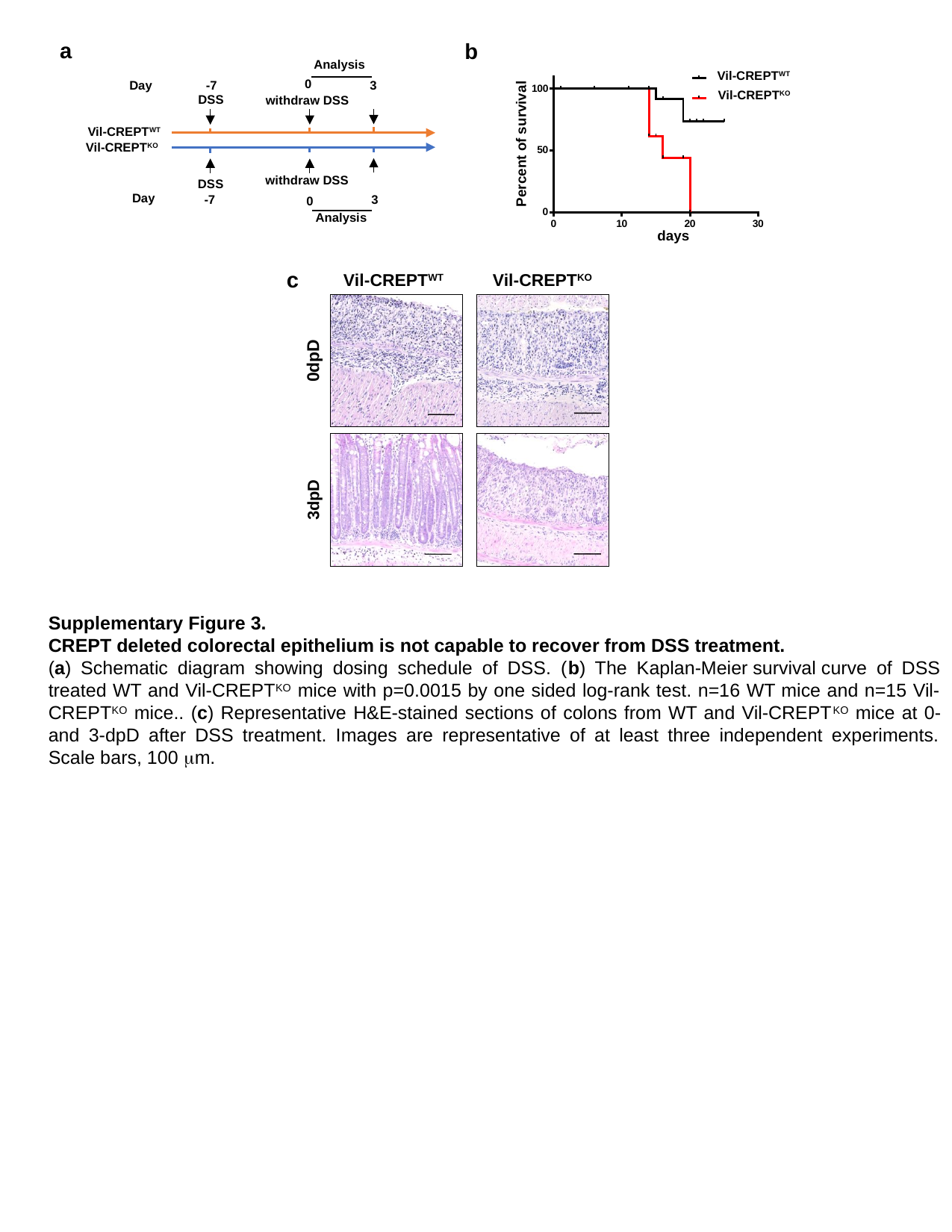

a
b
Analysis
Day
-7
DSS
Vil-CREPTWT
Vil-CREPTKO
DSS
Day
3
-7
0
Analysis
0
3
withdraw DSS
withdraw DSS
Vil-CREPTWT
Vil-CREPTKO
Percent of survival
days
c
Vil-CREPTWT
Vil-CREPTKO
0dpD
3dpD
Supplementary Figure 3.
CREPT deleted colorectal epithelium is not capable to recover from DSS treatment.
(a) Schematic diagram showing dosing schedule of DSS. (b) The Kaplan-Meier survival curve of DSS treated WT and Vil-CREPTKO mice with p=0.0015 by one sided log-rank test. n=16 WT mice and n=15 Vil-CREPTKO mice.. (c) Representative H&E-stained sections of colons from WT and Vil-CREPTKO mice at 0- and 3-dpD after DSS treatment. Images are representative of at least three independent experiments. Scale bars, 100 mm.

## Slide 6
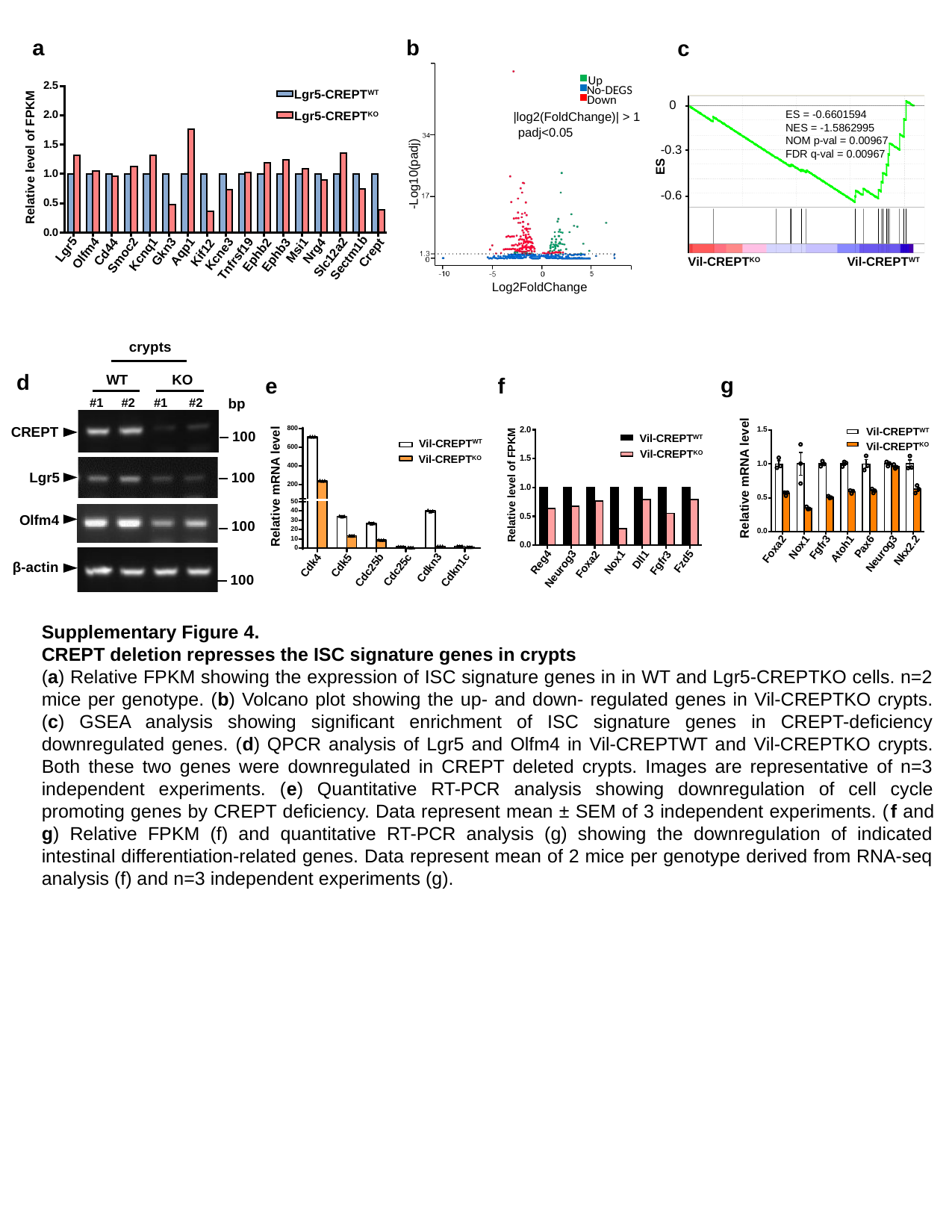

a
b
c
Up
No-DEGS
Down
|log2(FoldChange)| > 1
padj<0.05
-Log10(padj)
Log2FoldChange
Lgr5-CREPTWT
Lgr5-CREPTKO
Relative level of FPKM
Lgr5
Msi1
Nrg4
Aqp1
Gkn3
Cd44
Kif12
Crept
Olfm4
Kcnq1
Kcne3
Ephb2
Ephb3
Smoc2
Slc12a2
Sectm1b
Tnfrsf19
0
-0.3
-0.6
ES = -0.6601594
NES = -1.5862995
NOM p-val = 0.00967
FDR q-val = 0.00967
ES
Vil-CREPTKO
Vil-CREPTWT
crypts
WT
KO
CREPT
Lgr5
Olfm4
β-actin
#1
#2
#1
#2
bp
100
100
100
100
d
g
f
e
Vil-CREPTWT
Vil-CREPTKO
Relative mRNA level
Pax6
Nox1
Fgfr3
Atoh1
Foxa2
Nkx2.2
Neurog3
Vil-CREPTWT
Vil-CREPTKO
Relative level of FPKM
Dll1
Fzd5
Reg4
Nox1
Fgfr3
Foxa2
Neurog3
Relative mRNA level
Vil-CREPTWT
Vil-CREPTKO
Cdk4
Cdk5
Cdkn3
Cdc25c
Cdkn1c
Cdc25b
Supplementary Figure 4.
CREPT deletion represses the ISC signature genes in crypts
(a) Relative FPKM showing the expression of ISC signature genes in in WT and Lgr5-CREPTKO cells. n=2 mice per genotype. (b) Volcano plot showing the up- and down- regulated genes in Vil-CREPTKO crypts. (c) GSEA analysis showing significant enrichment of ISC signature genes in CREPT-deficiency downregulated genes. (d) QPCR analysis of Lgr5 and Olfm4 in Vil-CREPTWT and Vil-CREPTKO crypts. Both these two genes were downregulated in CREPT deleted crypts. Images are representative of n=3 independent experiments. (e) Quantitative RT-PCR analysis showing downregulation of cell cycle promoting genes by CREPT deficiency. Data represent mean ± SEM of 3 independent experiments. (f and g) Relative FPKM (f) and quantitative RT-PCR analysis (g) showing the downregulation of indicated intestinal differentiation-related genes. Data represent mean of 2 mice per genotype derived from RNA-seq analysis (f) and n=3 independent experiments (g).

## Slide 7
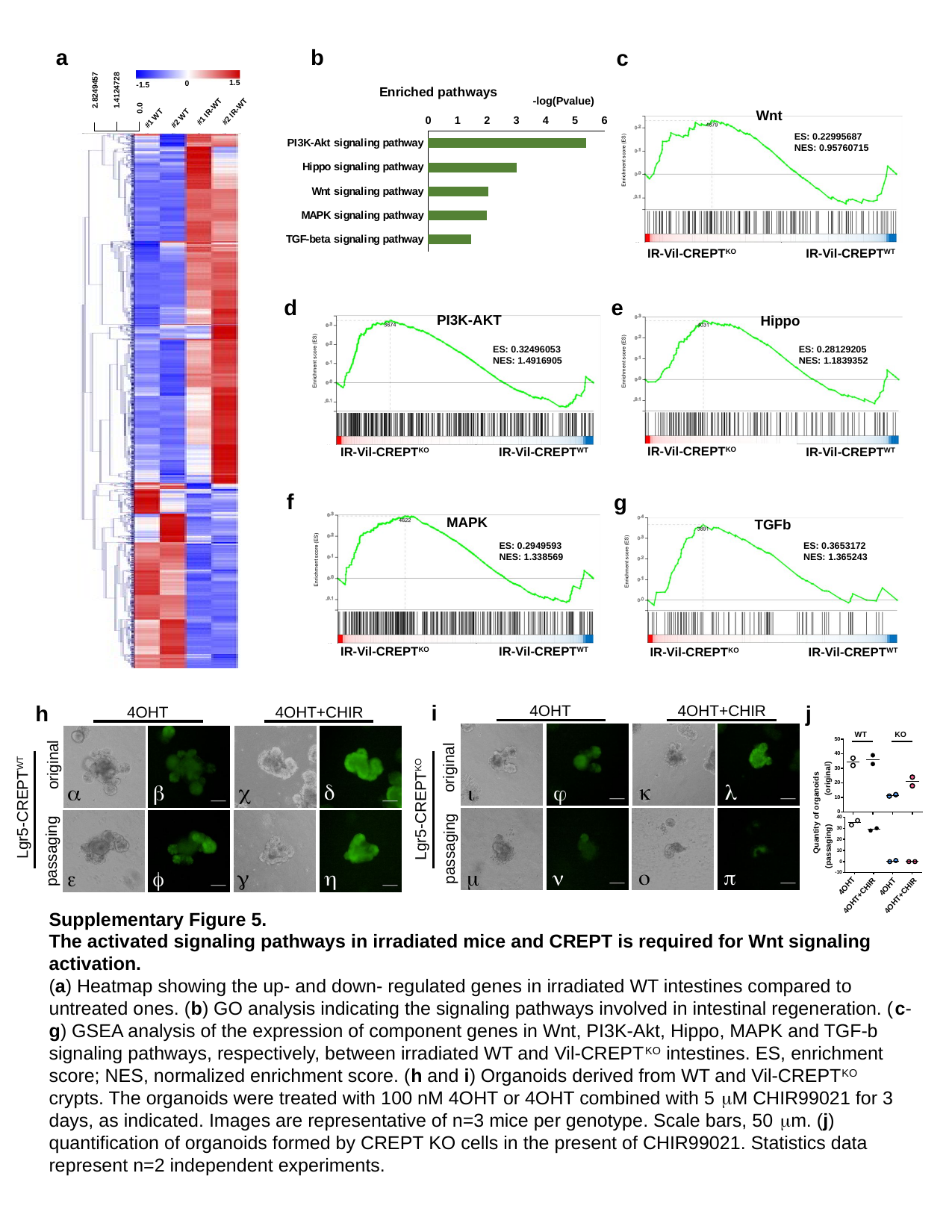

b
a
c
1.5
0
-1.5
2.8249457
1.4124728
0.0
#1 IR-WT
#2 IR-WT
#1 WT
#2 WT
### Chart: Enriched pathways
| Category | |
|---|---|
| PI3K-Akt signaling pathway | 5.376750709602099 |
| Hippo signaling pathway | 3.0132282657337552 |
| Wnt signaling pathway | 2.0604807473813813 |
| MAPK signaling pathway | 2.0 |
| TGF-beta signaling pathway | 1.4559319556497243 |-log(Pvalue)
Wnt
ES: 0.22995687
NES: 0.95760715
IR-Vil-CREPTKO
IR-Vil-CREPTWT
e
d
PI3K-AKT
Hippo
ES: 0.28129205
NES: 1.1839352
IR-Vil-CREPTKO
IR-Vil-CREPTWT
ES: 0.32496053
NES: 1.4916905
IR-Vil-CREPTKO
IR-Vil-CREPTWT
f
g
MAPK
ES: 0.2949593
NES: 1.338569
IR-Vil-CREPTKO
IR-Vil-CREPTWT
TGFb
ES: 0.3653172
NES: 1.365243
IR-Vil-CREPTKO
IR-Vil-CREPTWT
i
h
j
4OHT
4OHT+CHIR
4OHT
4OHT+CHIR
KO
WT
(original)
Quantity of organoids
(passaging)
4OHT
4OHT
4OHT+CHIR
4OHT+CHIR
original
original
k
l
c
d
i
j
a
b
Lgr5-CREPTWT
Lgr5-CREPTKO
passaging
passaging
o
p
g
h
m
n
e
f
Supplementary Figure 5.
The activated signaling pathways in irradiated mice and CREPT is required for Wnt signaling activation.
(a) Heatmap showing the up- and down- regulated genes in irradiated WT intestines compared to untreated ones. (b) GO analysis indicating the signaling pathways involved in intestinal regeneration. (c-g) GSEA analysis of the expression of component genes in Wnt, PI3K-Akt, Hippo, MAPK and TGF-b signaling pathways, respectively, between irradiated WT and Vil-CREPTKO intestines. ES, enrichment score; NES, normalized enrichment score. (h and i) Organoids derived from WT and Vil-CREPTKO crypts. The organoids were treated with 100 nM 4OHT or 4OHT combined with 5 mM CHIR99021 for 3 days, as indicated. Images are representative of n=3 mice per genotype. Scale bars, 50 mm. (j) quantification of organoids formed by CREPT KO cells in the present of CHIR99021. Statistics data represent n=2 independent experiments.

## Slide 8
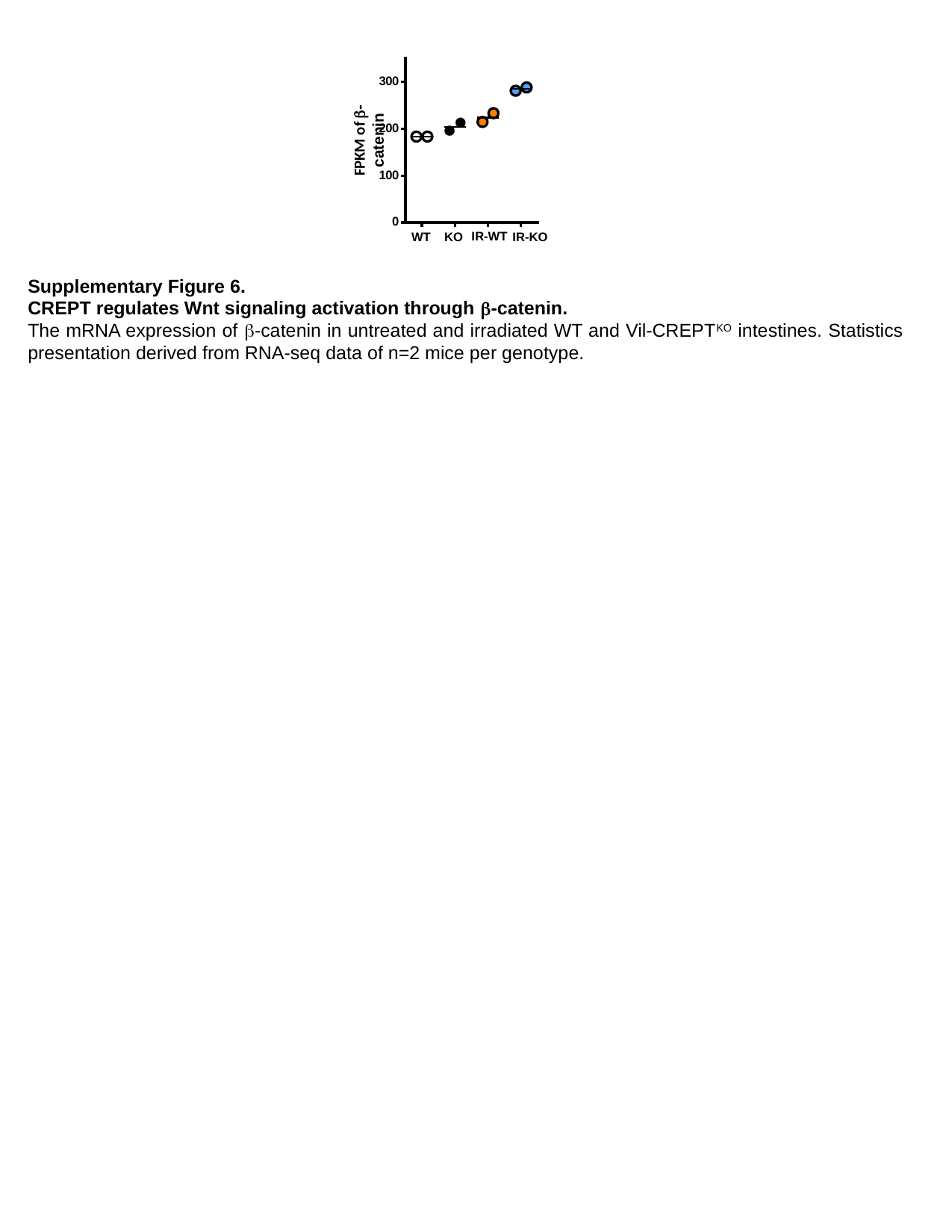

FPKM of b-catenin
IR-WT
KO
WT
IR-KO
Supplementary Figure 6.
CREPT regulates Wnt signaling activation through b-catenin.
The mRNA expression of b-catenin in untreated and irradiated WT and Vil-CREPTKO intestines. Statistics presentation derived from RNA-seq data of n=2 mice per genotype.

## Slide 9
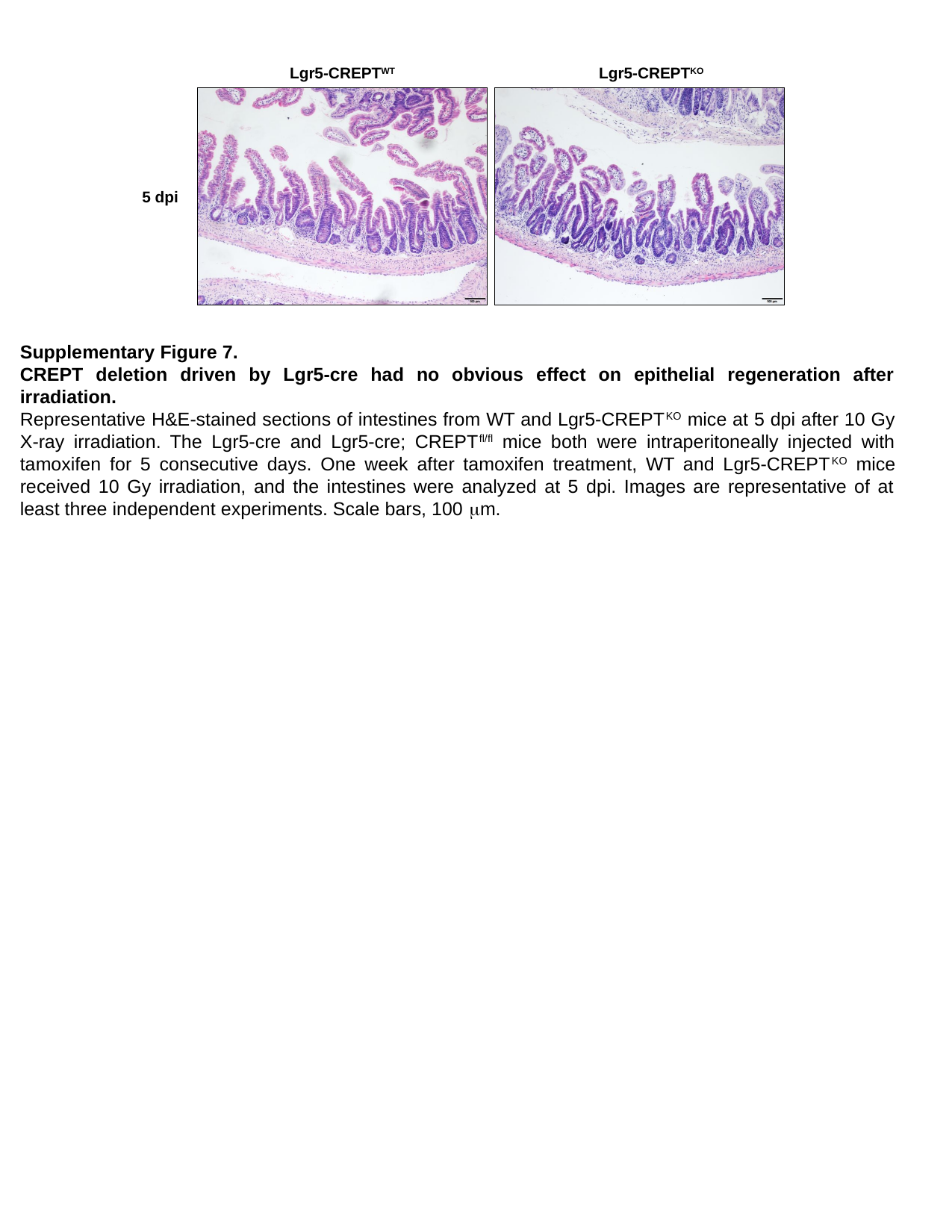

Lgr5-CREPTWT
Lgr5-CREPTKO
5 dpi
Supplementary Figure 7.
CREPT deletion driven by Lgr5-cre had no obvious effect on epithelial regeneration after irradiation.
Representative H&E-stained sections of intestines from WT and Lgr5-CREPTKO mice at 5 dpi after 10 Gy X-ray irradiation. The Lgr5-cre and Lgr5-cre; CREPTfl/fl mice both were intraperitoneally injected with tamoxifen for 5 consecutive days. One week after tamoxifen treatment, WT and Lgr5-CREPTKO mice received 10 Gy irradiation, and the intestines were analyzed at 5 dpi. Images are representative of at least three independent experiments. Scale bars, 100 mm.

## Slide 10
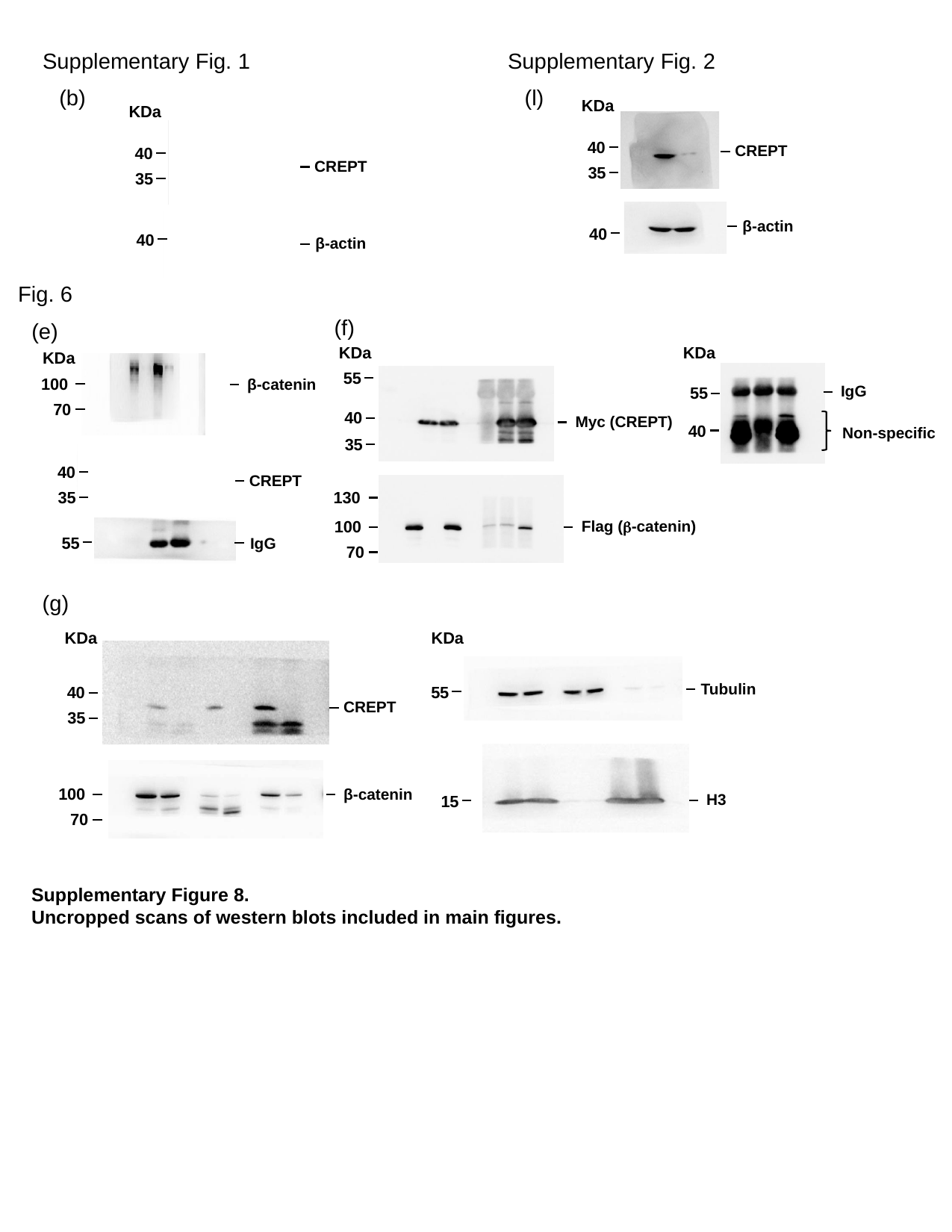

Supplementary Fig. 2
Supplementary Fig. 1
(l)
(b)
KDa
40
CREPT
35
β-actin
40
KDa
40
CREPT
35
40
β-actin
Fig. 6
(f)
(e)
KDa
55
40
Myc (CREPT)
35
130
100
Flag (b-catenin)
70
KDa
IgG
55
40
Non-specific
KDa
100
β-catenin
70
40
CREPT
35
55
IgG
(g)
KDa
40
CREPT
35
100
β-catenin
70
KDa
Tubulin
55
H3
15
Supplementary Figure 8.
Uncropped scans of western blots included in main figures.

## Slide 11
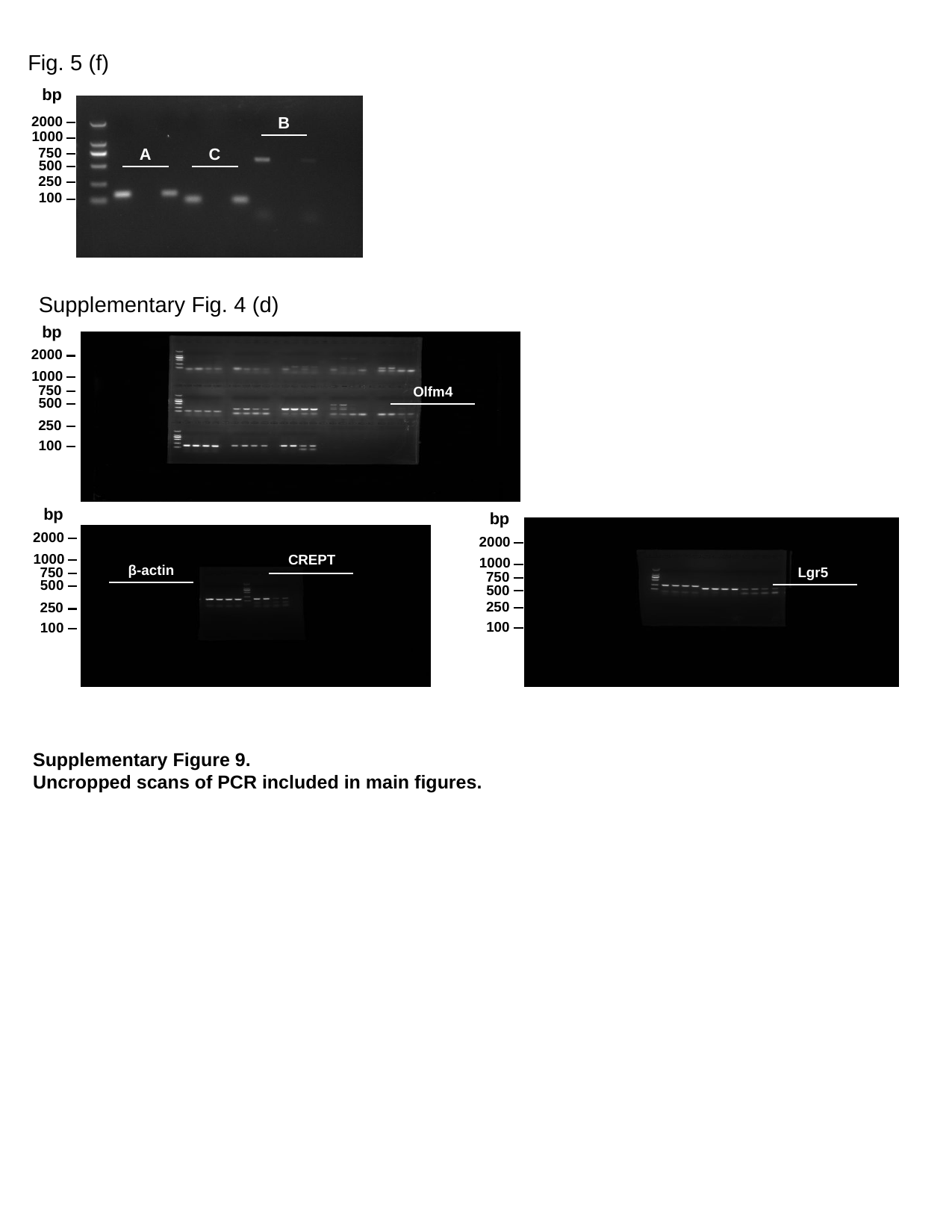

Fig. 5 (f)
bp
2000
B
1000
750
A
C
500
250
100
Supplementary Fig. 4 (d)
bp
2000
1000
750
Olfm4
500
250
100
bp
2000
1000
CREPT
β-actin
750
500
250
100
bp
2000
1000
Lgr5
750
500
250
100
Supplementary Figure 9.
Uncropped scans of PCR included in main figures.
