# Supplementary material for: CREPT is required for murine stem cell maintenance during intestinal regeneration
Source: Nat Commun. 2021 Jan 11;12:270. doi: 10.1038/s41467-020-20636-9 (PMC7801528; doi:10.1038/s41467-020-20636-9)
Supplement: Supplementary file 4 — Description of Additional Supplementary Files [file 41467_2020_20636_MOESM4_ESM.pdf]

Title: Supplementary Data 1

Description: PCR primers.
