# Supplementary material for: CREPT is required for murine stem cell maintenance during intestinal regeneration
Source: Nat Commun. 2021 Jan 11;12:270. doi: 10.1038/s41467-020-20636-9 (PMC7801528; doi:10.1038/s41467-020-20636-9)
Supplement: Supplementary file 5 — Reporting Summary [file 41467_2020_20636_MOESM5_ESM.pdf]

Corresponding author(s): Zhijie Chang

Last updated by author(s): Nov 28, 2020

Reporting Summary

Nature Research wishes to improve the reproducibility of the work that we publish. This form provides structure for consistency and transparency in reporting. For further information on Nature Research policies, see our Editorial Policies and the Editorial Policy Checklist.

Statistics

For all statistical analyses, confirm that the following items are present in the figure legend, table legend, main text, or Methods section.

n/a	Confirmed	
[ ]	[x]	The exact sample size (n) for each experimental group/condition, given as a discrete number and unit of measurement
[ ]	[x]	A statement on whether measurements were taken from distinct samples or whether the same sample was measured repeatedly
[ ]	[x]	The statistical test(s) used AND whether they are one- or two-sided<br>*Only common tests should be described solely by name; describe more complex techniques in the Methods section.*
[x]	[ ]	A description of all covariates tested
[x]	[ ]	A description of any assumptions or corrections, such as tests of normality and adjustment for multiple comparisons
[x]	[ ]	A full description of the statistical parameters including central tendency (e.g. means) or other basic estimates (e.g. regression coefficient) AND variation (e.g. standard deviation) or associated estimates of uncertainty (e.g. confidence intervals)
[ ]	[x]	For null hypothesis testing, the test statistic (e.g. F, t, r) with confidence intervals, effect sizes, degrees of freedom and P value noted<br>*Give P values as exact values whenever suitable.*
[x]	[ ]	For Bayesian analysis, information on the choice of priors and Markov chain Monte Carlo settings
[x]	[ ]	For hierarchical and complex designs, identification of the appropriate level for tests and full reporting of outcomes
[x]	[ ]	Estimates of effect sizes (e.g. Cohen's d, Pearson's r), indicating how they were calculated

Our web collection on statistics for biologists contains articles on many of the points above.

Software and code

Policy information about availability of computer code

Data collection	no software was used
Data analysis	no software was used

For manuscripts utilizing custom algorithms or software that are central to the research but not yet described in published literature, software must be made available to editors and reviewers. We strongly encourage code deposition in a community repository (e.g. GitHub). See the Nature Research guidelines for submitting code & software for further information.

Data

Policy information about availability of data

All manuscripts must include a data availability statement. This statement should provide the following information, where applicable:

- Accession codes, unique identifiers, or web links for publicly available datasets
- A list of figures that have associated raw data
- A description of any restrictions on data availability

The authors declare that all data supporting the findings of this study are available upon reasonable request.
The RNA-sequencing data reported in this study have been deposited in the Gene Expression Omnibus (GEO) database under accession codes: GSE143695 (https://www.ncbi.nlm.nih.gov/geo/query/acc.cgi?acc=GSE143695), GSE143604 (https://www.ncbi.nlm.nih.gov/geo/query/acc.cgi?acc=GSE143604) and GSE143605 (https://www.ncbi.nlm.nih.gov/geo/query/acc.cgi?acc=GSE143605). The ChIP-seq data have been deposited in the GEO database under accession code: GSE158243 (https://www.ncbi.nlm.nih.gov/geo/query/acc.cgi?acc=GSE158243).
The source data underlying Figs. 1-6 and Supplementary Figs. 2 and 4-6 are provided as a Source Data file.

Field-specific reporting

Please select the one below that is the best fit for your research. If you are not sure, read the appropriate sections before making your selection.

[x] Life sciences [ ] Behavioural & social sciences [ ] Ecological, evolutionary & environmental sciences

For a reference copy of the document with all sections, see nature.com/documents/nr-reporting-summary-flat.pdf

Life sciences study design

All studies must disclose on these points even when the disclosure is negative.

Sample size	All experiments reported in this study were repeated at least three independent times. For western blotting, immunohistochemistry, and immunofluorescence, representative images were derived from at least three animals per genotype. For Kaplan-Meier survival analysis, at least ten mice per genotype were involved.
Data exclusions	No data were excluded
Replication	Data were derived from at least three independent experiments.
Randomization	Samples were allocated into experiment group randomly.
Blinding	The investigators were blinded to group allocation during data collection and analysis.

Reporting for specific materials, systems and methods

We require information from authors about some types of materials, experimental systems and methods used in many studies. Here, indicate whether each material, system or method listed is relevant to your study. If you are not sure if a list item applies to your research, read the appropriate section before selecting a response.

Materials & experimental systems

n/a	Involved in the study	
[ ]	[x]	Antibodies
[ ]	[x]	Eukaryotic cell lines
[x]	[ ]	Palaeontology and archaeology
[ ]	[x]	Animals and other organisms
[x]	[ ]	Human research participants
[x]	[ ]	Clinical data
[x]	[ ]	Dual use research of concern

Methods

n/a	Involved in the study	
[ ]	[x]	ChIP-seq
[ ]	[x]	Flow cytometry
[x]	[ ]	MRI-based neuroimaging

Antibodies

Antibodies used	Antibodies included , rabbit anti-ki67 (Abcam, ab15580, 1:500), rabbit anti-Olfm4 (CST, 39141, 1:400), rabbit anti-Lysozyme (Dako, A0099, 1:1000), mouse anti-β-catenin (BD, 610154, 1:200), rabbit anti-cyclin D1 (Abcam, ab21699, 1:100), rabbit anti-GFP (CST, 2956, 1:200).
Validation	Mouse anti-CREPT antibody was validated in previously published paper (Ren.F, et al, 2014). Other antibodies were validated by manufacturers.

Eukaryotic cell lines

Policy information about cell lines

Cell line source(s)	National infrastructure of cell line resource of China
Authentication	STR of 293T cells: Amelogenin X CSF1PO 11 12 D13S317 12 D16S539 9 14 D18S51 17 18 D19S433 17 18 D21S11 28 30.2 D2S1338 19 D3S1358 5 16 17 D5S818 8 9 D7S820 11 12 D8S1179 12 13 14 FGA 21 22 23 TH01 7 9.3 TPOX 11 vWA 16 19 20.<br>STR of DLD-1 cells: Amelogenin X,Y CSF1PO 11,12 D12S391 19,22 D13S317 8,11 D16S539 12,13 D18S51 11,17 D19S433 14,16 D21S11 29,32.2 D2S1338 17,25 D3S1358 17,17 D5S818 13,13 D6S1043 11,13 D7S820 10,12 D8S1179 15,15 FGA 22,22 Penta E 7,14 TH01 7,9.3 TPOX 8,11 vWA 18,19.
Mycoplasma contamination	All cell lines were tested negative for mycoplasma contamination.

Commonly misidentified lines (See ICLAC register)	No misidentified line was used in the manuscript.

Animals and other organisms

Policy information about studies involving animals; ARRIVE guidelines recommended for reporting animal research

Laboratory animals	All mice were derived from C57BL/6J strain. Both male and female mice at 6-8 week were used.
Wild animals	The study did not involve any wild animal.
Field-collected samples	The study did not involve any sample collected from the field.
Ethics oversight	The laboratory animal facility has been accredited by AAALAC (Association for Assessment and Accreditation of Laboratory Animal Care International) and the IACUC (Institutional Animal Care and Use Committee) of Tsinghua University approved all animal protocols used in this study.

Note that full information on the approval of the study protocol must also be provided in the manuscript.

ChIP-seq

Data deposition

- [x] Confirm that both raw and final processed data have been deposited in a public database such as GEO.
- [x] Confirm that you have deposited or provided access to graph files (e.g. BED files) for the called peaks.

Data access links *May remain private before publication.*	GSE158243 (https://www.ncbi.nlm.nih.gov/geo/query/acc.cgi?acc=GSE158243)
Files in database submission	GSE158243_CREPT.wiggle.tar.gz
Genome browser session (e.g. UCSC)	http://www.igv.org/

Methodology

Replicates	No replicate was involved in ChIP-seq data, but ChIP was performed to validate the results in the manuscript.
Sequencing depth	28105125 clean reads with 1,351,753,500 bp, 90% mapping rate.
Antibodies	mouse anti-CREPT antibody (Ren.F, et al, 2014)
Peak calling parameters	Clean reads were mapped to GCF_000001635.26_GRCm38.p6 genome sequence from NCBI using SOAPaligner/soap2. Peak calling was performed by MACS.
Data quality	1257 Peak Number, 187485 Total Length, 149 Average Length, 11764 Total Tag Depth, 9 Average Tag Depth
Software	FastQC, soap2, Bowtie2, MACS

Flow Cytometry

Plots

Confirm that:

- [x] The axis labels state the marker and fluorochrome used (e.g. CD4-FITC).
- [x] The axis scales are clearly visible. Include numbers along axes only for bottom left plot of group (a 'group' is an analysis of identical markers).
- [x] All plots are contour plots with outliers or pseudocolor plots.
- [x] A numerical value for number of cells or percentage (with statistics) is provided.

Methodology

Sample preparation	The intestinal epithelial cells were disaggregated using EDTA in PBS. The supernatant containing villi and crypts passed through 70m cell strainers (Corning) to collect crypts. The crypts were then suspended in TryPLE (Gibco, 12604) to be disaggregated into single cells. The disaggregated cells were passed through 40 um cell strainers (Corning) and analyzed by flow cytometry (BD Arial)
Instrument	BD Arial

Software	Flowjo
Cell population abundance	The total crypt cells had about 10% Lgr5-GFP positive cells. The sorted cells were validated by RT-qPCR to confirm the Lgr5 expression in Lgr5-GFP negative and positive cells.
Gating strategy	Using the GFP negative crypt cells as control, the Lgr5-GFP positive cells were gated.

☒ Tick this box to confirm that a figure exemplifying the gating strategy is provided in the Supplementary Information.
